# Supplementary material for: Genomic Variations in Susceptibility to Intracranial Aneurysm in the Korean Population
Source: J Clin Med. 2019 Feb 25;8(2):275. doi: 10.3390/jcm8020275 (PMC6406302; doi:10.3390/jcm8020275)
Supplement: Supplementary file 1 [file jcm-08-00275-s001.pdf]

Supplementary data

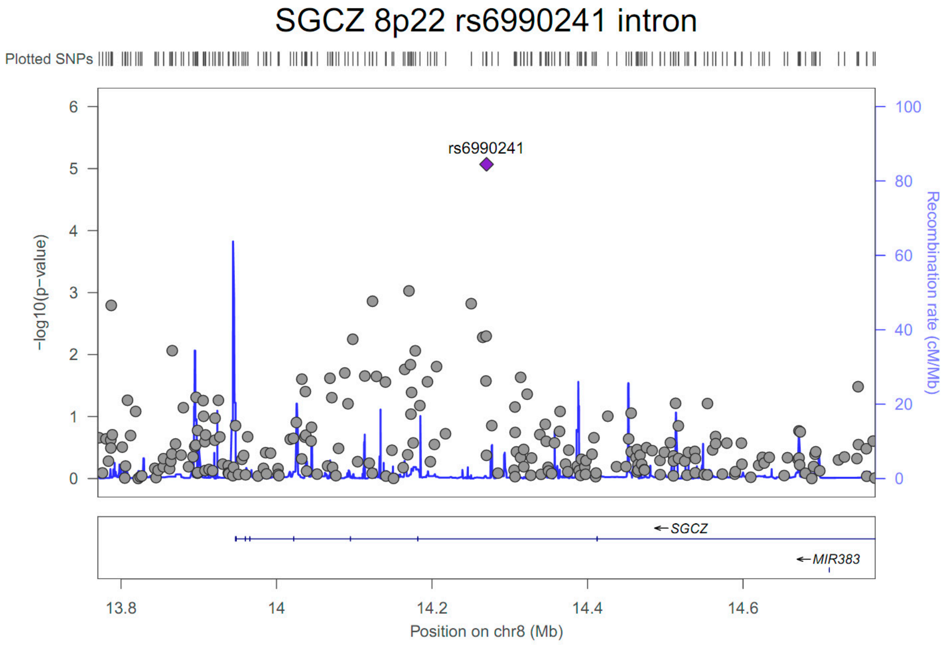

Figure S1. A regional plot of the rs6990241 (intron, *SGCZ*, 8p22) variant's position ± 500kb demonstrates associations with subarachnoid hemorrhage in 250 patients with intracranial aneurysm after adjusting for age, sex, hypertension, diabetes, hyperlipidemia, cigarette smoking, and four principal components. The X- and Y-axes indicate the chromosomal position (mega base, Mb) and $-\log_{10}$ transformed *P*-values, respectively. The purple diamond indicates the rs6990241 variant demonstrating suggestive association in our genome-wide association study ($P = 8.5 \times 10^{-6}$). The gray circles indicate other SNPs within the rs6990241 variant's position ± 500kb.

Table S1. Associations of 17 previously reported SNPs with intracranial aneurysms in our genome-wide association study.

Gene	Chr.	SNP	Class	M/m[a]	MAF[b]	OR[c]	*P*[c]	Asian ethnicity	Reference (First author)
COL3A1	2q32.2	rs1800255	A698T	G/A	0.23/0.27	0.94	0.670	CHI	Chen, Zhu
BOLL	2q33.1	rs700651	intron	A/G	0.48/0.45	1.42	0.008	JPT	Bilguvar, Hashikata, Low
EDNRA	4q31.22	rs6841581	Near UTR-5	G/A	0.13/0.22	0.53	6.5E-04	JPT	Yasuno
LOX	5q23.2	rs1800449	R158L	C/T	0.19/0.20	0.97	0.849	KOR	Hong
FBN2	5q23.3	rs331079	intron	G/C	0.04/0.04	0.86	0.646	JPT	Ruigrok
ELN	7q11.23	rs2071307	G422S	G/A	0.13/0.14	0.73	0.117	CHI, KOR	Yang, Jeon
NOS3	7q36.1	rs1799983	D298E	G/T	0.10/0.08	1.18	0.482	JPT,IND, KOR	Krischek, Koshy, Kim
RNU105C	8q11.23	rs10958409	intergenic	G/A	0.22/0.24	1.04	0.781	JPT	Bilguvar, Hashikata, Low, Akiyama
SOX17	8q11.23	rs9298506	intergenic	A/G	0.21/0.22	0.98	0.902	JPT	Bilguvar, Low, Yasuno
CDKN2B-AS1	9p21.3	rs1333040	intron	T/C	0.33/0.31	1.05	0.746	JPT	Bilguvar, Hashikata, Low, Akiyama, Yasuno, Nakaoka
CDKN2B-AS1	9p21.3	rs10757272	intron	T/C	0.36/0.33	1.07	0.629	JPT	Low
CDKN2B-AS1	9p21.3	rs2891168	intron	A/G	0.47/0.46	1.10	0.445	JPT	Nakaoka
CDKN2B-AS1	9p21.3	rs10757278	intergenic	A/G	0.49/0.47	1.13	0.328	JPT	Hashikata, Nakaoka
FGD6	12q22	rs6538595	intron	G/A	0.37/0.34	1.03	0.856	JPT	Yasuno
SERPINA3	14q32.13	rs4934	A9T	A/G	0.37/0.41	0.80	0.087	JPT, CHI	Krischek, Liu
MMP2	16q12.2	rs243865	intron	C/T	0.12/0.12	0.92	0.693	JPT	Low
CTAGE1	18q11.2	rs11661542	intergenic	C/A	0.37/0.42	0.89	0.383	JPT	Low, Yasuno

Chr., chromosome; CHI, Chinese; IND, Indian; KOR, Korean; JPT, Japanese; OR, odds raito; [a] M/m, major/minor allele type; [b] MAF, minor allele frequency in case (left) and control (right); [c] OR, 95% confidence interval, and p-value were estimated in the multivariate logistic regression model after adjustment for age, sex, hypertension, diabetes, hyperlipidemia, cigarette smoking, and four principal components.

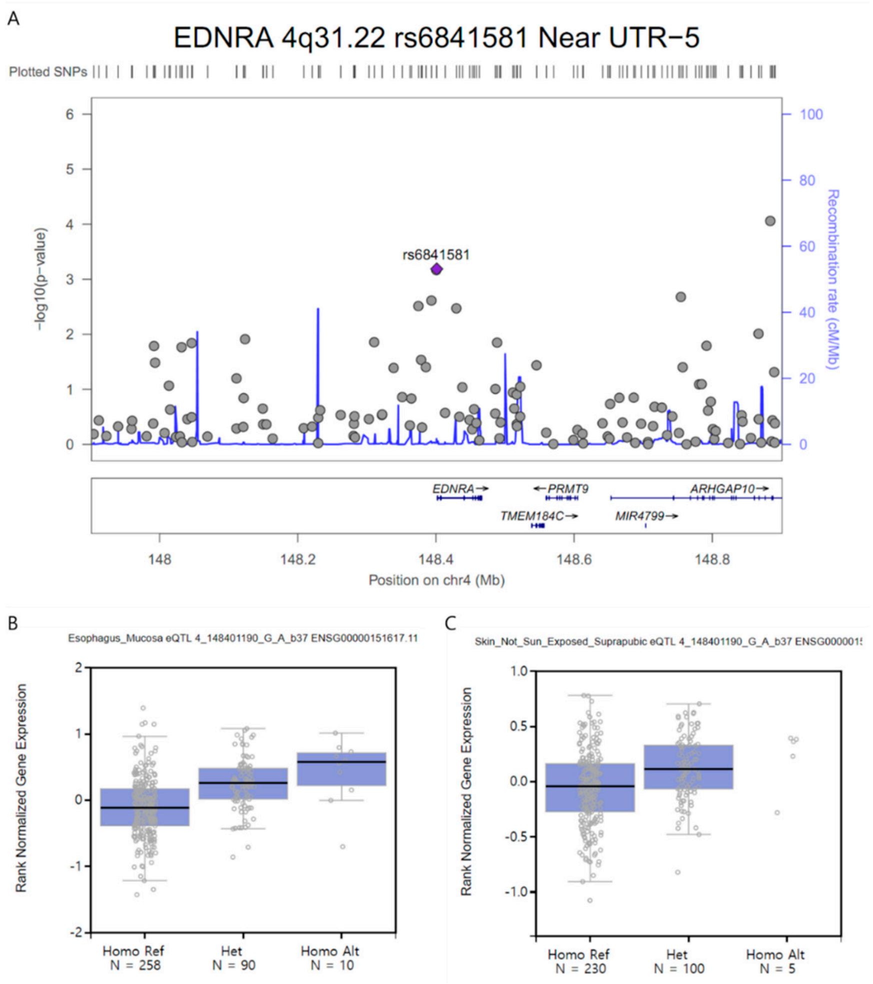

Figure S2. (A) A regional plot of (A) the reported SNP, rs6841581 (near UTR-5, *EDNRA*, 4q31.22) variant's position ± 500kb demonstrating a replicated association with intracranial aneurysms in 250 patients and 294 controls after adjusting for age, sex, hypertension, diabetes, hyperlipidemia, cigarette smoking, and four principal components. The X- and Y-axes indicate the chromosomal position (mega base, Mb) and $-\log_{10}$ transformed *P*-values, respectively. The purple diamond indicates the rs6841581 variant replicated in our genome-wide association study ($P = 6.5\times10^{-4}$). The gray circles indicate other SNPs within the rs6841581 variant's position ± 500kb. The expression level of the rs6841581 variant in (B) the esophagus (mucosa) (n = 358, effect size = 0.38, $P = 5.2\times10^{-12}$) and (C) the skin not exposed to the sun (suprapubic) (n = 335, effect size = 0.21, $P = 1.2\times10^{-6}$) tissues, respectively, were measured by analyzing the single-cell expression quantitative trait loci (eQTL) in GTeX portal (https://gtexportal.org/home). The X- and Y-axes indicate three genotypes for each variant and the rank-normalized gene expression levels, respectively. The Reference (Ref)/Alternative (Alt) alleles of each variant's genotypes are equal to their major/minor alleles, respectively. Homo, homozygote; Het, heterozygote.

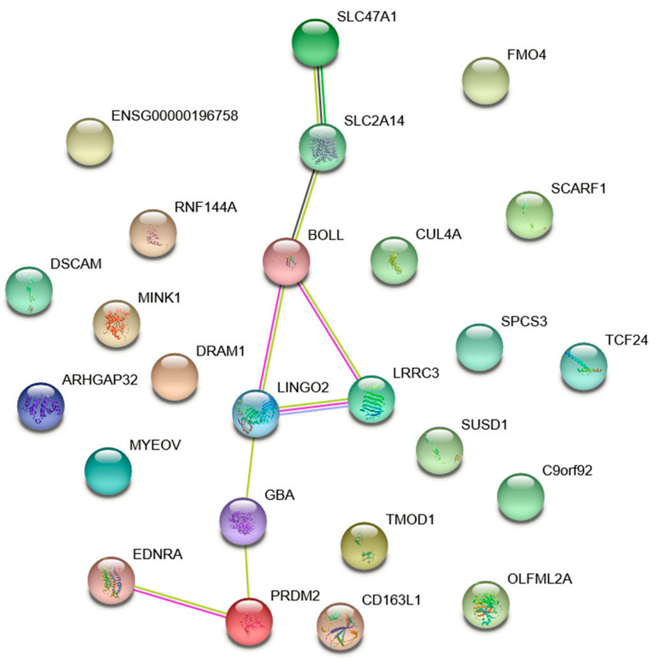

Figure S3. Shown here is a network of multiple protein-coding gene-gene interactions using 25 out of 31 candidate genes for susceptibility to intracranial aneurysms (six genes, *LINC01237*, *LINC00474*, *LINC02130*, *LOC102724084*, *NAPA-AS1*, and *SLC5A4-AS1*, were not available in the STRING v.10.5 database; ENSG00000196758 indicates the *FLJ45964* gene) (*Homo sapiens*). Individual's edges (lines) indicate associations between genes (i.e., genes jointly contribute to a shared function): light green lines indicate co-publication in PubMed (text mining); light purple indicates evidence of homology interacting in other species; purple lines indicate experimental evidence for interaction between genes; black lines indicate evidence of mRNA co-expression of the associated genes; and light blue indicates evidence for association from pathway database.
